# Total Chemical Synthesis of LC3A and LC3B Activity-Based Probes

**DOI:** 10.3390/biomedicines11030884

**Published:** 2023-03-13

**Authors:** Yara Huppelschoten, Jens Buchardt, Thomas E. Nielsen, Aysegul Sapmaz, Gerbrand J. van der Heden van Noort

**Affiliations:** 1 Department of Cell and Chemical Biology, Leiden University Medical Centre, 2333 ZC Leiden, The Netherlands; 2 Novo Nordisk A/S, Global Research Technologies, Novo Nordisk Park, DK-2760 Måløv, Denmark; 3 Novo Nordisk A/S, CMC API Development, DK-2880 Bagsværd, Denmark

**Keywords:** activity-based probe, chemical synthesis, protein chemistry

## Abstract

Autophagy is a conserved cellular process involved in the degradation of intercellular materials. During this process, double-membrane vesicles called autophagosomes engulf cytoplasmic components ready for degradation. A key component in the formation of autophagosomes are the autophagy-related (Atg) proteins, including microtubule-associated protein light chain 3A (LC3A) and 3B (LC3B). After the C-terminus of LC3 is conjugated to a phospholipid, it promotes the elongation of the phagosome and provides a docking station for the delivery of proteins ready for degradation. Since dysregulation of the autophagy pathway has been associated with a variety of human diseases, components of this process have been considered as potential therapeutic targets. However, the mechanistic details of LC3-specific ligases and deconjugation enzymes are far from unraveled and chemical tools for activity profiling could aid in affording more insights into this process. Herein, we describe a native chemical ligation approach for the synthesis of two LC3 activity-based probes (ABPs). Initial studies show that the probes covalently interact with the cysteine protease ATG4B, showcasing the potential of these probes to unravel mechanistic and structural details.

## 1. Introduction

**Figure 1:**
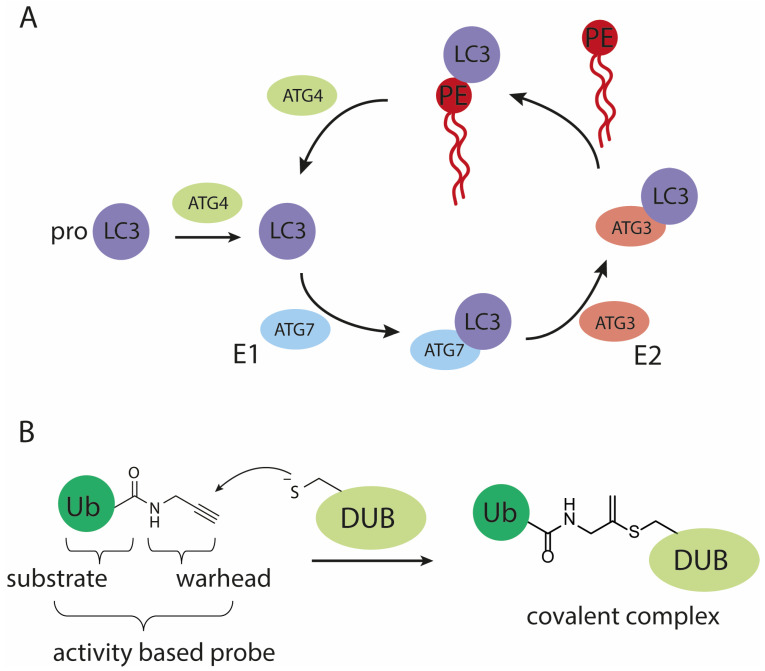
Schematic representation of (**A**) the (de)conjugation machinery involved in maturing and processing LC3, (**B**) the mode of action of an active cysteine deubiquitylating protease (DUB) on a propargylamide activity-based probe.

Autophagy is a catabolic process for the bulk degradation of intercellular materials, such as damaged organelles, long-lived and aggregated proteins. These cytoplasmic components are engulfed in double-membrane vesicle structures called autophagosomes upon stress or nutrient deprivation. Subsequent autophagosome maturation is achieved by the fusion with endosomes and/or lysosomes to form autolysosomes, whereafter the cargo is degraded by lysosomal hydrolases [1,2]. Dysfunction of autophagy has been associated with a number of diseases, including neurodegeneration, cancer and pathogenic infection [1,3]. For these reasons, there has been a growing interest in the still enigmatic mechanism of autophagy at molecular level. Key components in the autophagy process are the autophagy-related (Atg) proteins. In mammals, there are at least six Atg protein orthologs: microtubule-associated protein light chain 3 A (LC3A), B (LC3B), C (LC3C), GABA type A receptor-associated protein (GABARAP), GABARAP-like 1 (GABARAP1) and GABARAP-like 2 (GABARAP2) [4]. Functional characterization has been mostly focused on the LC3 paralogs since their discovery, and they have been widely used as a marker for autophagosomes and to measure autophagy activity [5,6]. During the process of autophagy, LC3 is connected to phosphatidylethanolamine (PE) via an amide bond with its C-terminal glycine and subsequently obtains correct membrane localization and function. PE-conjugation to LC3 is directed by two proteins, where in the first step mature LC3 is obtained by autophagy-regulating protease 4 (Atg4)-mediated cleavage of the C-terminal residues of the immature precursor pro-LC3. In the second step, mature LC3 serves as a substrate in the conjugation reaction catalyzed by Atg7 (E1-like activity) and Atg3 (E2-like activity), to allow conjugation to PE (Figure 1A) [7]. The resulting lipid-anchored protein promotes the elongation of the phagosome and provides a docking station for receptor proteins to deliver cytoplasmic materials targeted for degradation. Afterwards, LC3 is either enzymatically released from PE and thereby the membrane, by Atg4 or degraded by the lysosomal proteases [8]. Up to date, LC3’s biological function remains enigmatic and difficult to study due to the lack of proper tools. In many fields, including (de)ubiquitylation [9,10,11], (de)SUMOylation [12] and (de)UFMylation [13], activity-based probes (ABPs) have been helpful in identifying proteins involved in these dynamic post-translational modifications and contributed in elucidating some of the molecular details of such systems. ABPs are powerful chemical tools that mimic the natural substrates of conjugation and deconjugation enzymes. The crucial difference with natural substrates is that they are equipped with a reactive group, often referred to as warhead, that reacts with the active site cysteine (in the case of cysteine-protease/ligase enzymes) to form an irreversible covalent bond (Figure 1B) [14,15,16,17]. In analogy to the reported ubiquitin (Ub)-, small ubiquitin-related modifier (SUMO)- and ubiquitin-fold modifier 1 (UFM1)-tools we deemed a reliable route towards the chemical synthesis of LC3 and ABPs essential to facilitate a detailed investigation of LC3 functioning in autophagy.

Pioneering preparation of LC3B and analogues based thereon rely on semi-synthetic methods such as expressed protein ligation (EPL) making use of an expressed N-terminal region of LC3B, that is further modified with small chemically prepared C-terminal peptides or lipids [18,19,20]. A limitation in this approach is that the introduction of chemical entities to modify proteins on the N-terminus such as fluorescent tags or affinity handles and non-naturally occurring amino acid mutations is difficult and therefore chemical synthesis offers an attractive alternative. In addition to the semi-synthetic approaches, we recently described the total linear chemical synthesis of LC3A and LC3B [21] that in analogy to Ub allows for a straightforward incorporation of unnatural amino acids or functional groups at will. C-terminal modifications such as warheads to covalently capture active site cysteines of proteases, however, cannot be incorporated using this methodology. The linear synthesis we reported employs synthesis at an elevated temperature to optimize amino acid-coupling efficiency and improve overall yield and hence is incompatible with the acid labile 2-chlorotrityl chloride (CTC) resin often used to introduce C-terminal modifications. To redeem this issue, we here present a more practical route using a two-segment native chemical ligation (NCL) approach towards full-length LC3A and LC3B activity-based probes (ABPs), allowing both N-terminal and C-terminal modifications simultaneously. This synthetic strategy opens possibilities for the future synthesis of valuable tools that can be applied in the characterization of enzymatic activities which could lead to more insight into the dynamics of the LC3 (de)conjugation machinery during autophagy.

## 2. Materials and Methods

### 2.1. Solid Phase Peptide Synthesis

#### 2.1.1. Automated Fmoc Solid Phase Peptide Synthesis

Solid Phase Peptide Synthesis (SPPS) was performed on a Symphony X (Gyros Protein Technologies, Uppsala, Sweden) automated peptide synthesizer using 9-fluorenylmethoxycarbonyl (Fmoc) as N-terminal protective group on the respective amino acids. The Fmoc group was removed using 2 × 10 min treatments with 20 vol. % piperidine, 0.1% Oxyma Pure^®^ in dimethylformamide (DMF). N,N′-Diisopropylcarbodiimide (DIC)/Oxyma (0.3 M/0.3 M in DMF) was used as the activator in peptide couplings with 4–6-fold excess together with equal equivalents of DIC (1.5 M in DMF). The coupling time was 2 h unless specified otherwise. All dipeptide building blocks were coupled for 4 h. The residual free amino groups after the coupling reaction were capped by the addition of collidine (3.3 equivalents, 1.5 M in DMF) and acetic anhydride (11 equivalents, 1.0 M in DMF) and were reacted for 20 min. Final Fmoc deprotection was followed by washing with DMF and dichloromethane (DCM).

#### 2.1.2. Global Deprotection and Resin Release

The peptide sequences containing a cysteine residue were released from the resin and protective groups were removed simultaneously with Reagent K (trifluoroacetic acid (TFA)/phenol/H_2_O/thioanisole/ethane-1,2-dithiol (EDT), 82.5:5:5:5:2.5 *v*/*v*/*v*/*v*/*v*) for 2 h followed by precipitation in diethylether (0 °C) and collected by centrifugation. Peptides that contain a methionine residues in the sequence were detached from the resin and deprotected using TFA/tri-iso-propylsilane (TIPS)/H_2_O/DCM/NH_4_I/dithiothreitol (DTT), 87:5:2.5:2.5:0.5:2.5, *v*/*v*/*v*/*v*/*v*/*v* for 2–3 h followed by precipitation in diethylether (0 °C) and collected by centrifugation. The pellet was resuspended in diethylether before being collected by centrifugation again. The precipitate was subsequently dissolved in H_2_O/CH_3_CN/acetic acid (AcOH), 65:25:10, *v*/*v*/*v* and lyophilized before purification.

#### 2.1.3. Purification of Peptides

The peptides were purified on a preparative Gilson HPLC system (Gilson Inc., Middleton, WI, USA) using a reversed phase HPLC column (Phenomenex Inc., Torrance, CA, USA) as specified in the supporting experimental section. Two mobile phases were used for elution: A = 0.1% TFA in deionized water (Veolia, Saint-Maurice, France) and B = 0.1% TFA in acetonitrile using a linear gradient. Relevant fractions were selected by analytical LC-MS (Waters Acquity H-class HPLC coupled to LCT premier micromass spectrometer; Waters Corp., Milford, MA, USA) and fractions containing the pure peptide were pooled and lyophilized.

#### 2.1.4. Synthesis of Peptide 4

The synthesis was performed following the procedure for automated SPPS described in the Supplementary Materials using Chemmatrix resin (Novabiochem, Merck Millipore, Darmstadt, Germany), preloaded with 3,4-diaminobenzoic acid allyloxycarbonyl (Dbz(Alloc)) (0.89 g, 0.23 mmol/g). Allyloxycarbonyl (Alloc) deprotection was achieved by swelling the resin in dry DCM and adding Pd(PPh_3_)_4_ (83 mg, 0.07 mmol, 0.35 equivalent) and morpholine (356 µL, 4 mmol, 20 equivalents). The reaction proceeded for 4 h and afterwards the resin was washed with DMF (3 × 2 mL for 1 min) and DCM (3 × 2 mL for 1 min). The peptide was cleaved from the resin according to the procedure described above in global deprotection and resin release. Peptide 2 was dissolved in 6 M guanidinium (Gdn). HCl pH 3.0 (1 mM final concentration) and 1 M NaNO_2_ in deionized water (2.0 mL, 2.0 mmol, 10 equivalents) was added and stirred for 5 min at 0 °C. The reaction was warmed to room temperature and sodium 2-mercaptoethanesulfonate (MESNa) (3.49 g, 20.5 mmol, 100 equivalents) in 6 M Gdn.HCl, 0.2 M NaH_2_PO_4_ pH 7.0 was added. The pH was adjusted to pH 7.0 and the solution was stirred for 20 min before purification by preparative HPLC using a Gemini^®^ (Phenomenex Inc., Torrance, CA, USA) 110 Å, C18, 5 μm, 30 mm × 250 mm column (25 to 35% B over 60 min, 30 mL/min). Lyophilization afforded peptide 4 as a white solid (16.6 mg, 0.83% yield), calculated mass: 9884.8 Da, observed: 9885.0 Da.

#### 2.1.5. Synthesis of Peptide 9

The synthesis was performed following the procedure for automated SPPS described in the Supplementary Materials using 2-chlorotrityl resin preloaded with Fmoc-Glycine (0.41 g, 0.42 mmol/g). As final step, the Belyntic (Belyntic GmbH, Berlin, Germany) linker (2-((2-(2-bis-(tert-butoxycarbonyl)-(aminooxy)acetamido)ethyl)carbamoyl)-4-azido-3-bromo benzyl(4-nitrophenyl) carbonate) was coupled according to the manufacturer’s protocol. The protected polypeptide was detached from the resin by treatment with hexa-fluoro-iso-pro panol (HFIP)/DCM (1:3), 3× for 15 min. All filtrates were combined and concentrated under reduced pressure using a rotation film evaporator (Buchi Labortechnik AG, Flawil, Switzerland), followed by co-evaporation of the protected protein by dichloroethane (DCE). Subsequently, the protected protein was dissolved in DCM and propargylamine (4 equivalents, 44 µL, 0.68 mmol) and di-isopropyl-ethylamine (DIPEA) (2 equivalents, 59 µL, 0.34 mmol) were added and reacted for 16 h. The solvents were removed in vacuo using a rotation film evaporator and the protecting groups were cleaved according to the procedure described above for global deprotection and resin release. The crude peptide was purified by following the procedure from Belyntic [22]. Lyophilization afforded peptide **9** as a white solid (12.9 mg, 1.5% yield), calculated mass: 4891.2 Da, observed: 4891.7 Da.

### 2.2. Assembly of Biotin-LC3B-PA (11)

Peptide 4 (18.7 mg, 0.0020 mmol) and peptide 9 (12.1 mg, 0.0025 mmol) were dissolved in 6 M Gdn.HCl/0.2 M NaH_2_PO_4_, pH 7.2 at a final concentration of 1 mM. Mercaptophenylacetic acid (MPAA) and tris(2-carboxyethyl)phosphine (TCEP) were added from a 1 M stock in deionized water to a final concentration of 100 mM and 25 mM. Then, pH was adjusted to 7.0 and the reaction was shaken for 16 h at 37 °C upon which LC-MS analysis (Waters Acquity H-class HPLC coupled to a LCT premier micromass spectrometer; Waters Corp., Milford, MA, USA) showed that the reaction was complete. The crude peptide was purified by preparative HPLC using a Gemini^®^ (Phenomenex Inc., Torrance, CA, USA) 110 Å, C4, 5 μm, 10 mm × 250 mm column (25 to 45% B over 20 min, 5 mL/min). Lyophilization afforded peptide 11 as a white solid (2.59 mg, 8.9% yield), calculated mass: 14,715.1 Da, observed: 14,716.0 Da.

### 2.3. Pull-Down of Overexpressed GFP-ATG4B

Human Hek293T (Cat#ATCC^®^ CRL-3216™) cell line purchased from ATCC (Manassas, VI, USA) was cultured in Dulbecco’s modified Eagle’s medium (DMEM) (Gibco) supplemented with 7.5% Fetal Calf Serum (FCS) and was maintained at 37 °C and 5% CO_2_. HEK293T cells expressing GFP, GFP-ATG4B or GFP-ATG4B C74A mutant were lysed in 300 μL lysis buffer (0.8% NP40, 150 mM NaCl, 50 mM Tris-HCl pH 8.0, 0.05 mM MgCl_2_ plus cOmplete, EDTA-free protease inhibitor cocktail (Roche; Cat# 05056489001) followed by brief sonication. Cell debris was removed by centrifugation. Next, 3 μM final concentration of biotin-tagged synthetic LC3B-PA (11) or LC3A-PA (12) were added to cell lysates of GFP-, GFP-ATG4B- and GFP-ATG4B C74A-expressing cells and incubated for 1 h at 37 °C. Thereafter, the reaction volume was completed to 1 mL and 30 μL of high capacity NeutrAvidin agarose beads (Thermo Fisher Scientific; Cat# 29202; Waltham, MA, USA) were added incubated by rotating for overnight at 4 °C. The beads were extensively washed with lysis buffer (0.8% NP40, 150 mM NaCl, 50 mM Tris-HCl pH 8.0, 0.05 mM MgCl_2_) containing 2% SDS for 5 times. After completely removing the washing buffer, the SDS sample buffer supplemented with 2-mercaptoethanol was added to the beads and boiled at 95 °C. The proteins were separated by SDS-PAGE followed by Western blotting using rabbit anti-GFP antibody (homemade, A996, dilution 1:1000). Secondary IRDye 800CW goat anti-rabbit IgG (H + L) (Li-COR Inc., Lincoln, NE, USA, Cat# 926-32211, dilution 1:20,000) was used for detection using the Odyssey Classic imager (Li-COR).

## 3. Results

Deubiquitinating proteases (DUBs) are effectively targeted by Ub-probes carrying a propargylamide (PA) warhead, showing exquisite selectivity for active-site cysteine residues of DUBs over other proteases [14]. Applying this warhead in probes that target proteases that act on SUMO (sentrin specific proteases: SENPs) [12] or UFM1 (UFM1 specific proteases: UFSPs) [13] was also successful and hence we envisioned that a propargyl probe would be a good choice to target proteases involved in pro-LC3 maturation and or LC3-PE proteolysis. In addition to this C-terminal modification, a biotin tag (Bt) was introduced at the N-terminus to allow enrichment strategies via biotin–streptavidin interactions.

**Figure 2:**
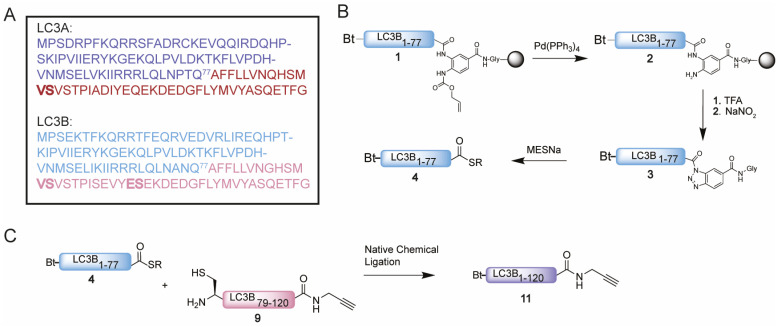
(**A**) Sequence of LC3A and LC3B with Gln77 as site of NCL and N-terminal peptide and C-terminal peptide in blue and magenta, respectively. Pseudoproline dipeptides introduced after automated fast flow peptide synthesis (AFPS) analysis are indicated in bold. (**B**) Schematic representation of the synthesis of thioester 4. (**C**) Schematic representation of the NCL reaction between thioester 4 and cysteine peptide 9 towards Bt-LC3B-propargylamide 11.

We started with the examination of the primary amino acid sequence of LC3A and LC3B to identify potential sites that could be used for the connection of two shorter peptide segments using native chemical ligation. LC3A and LC3B share a high sequence similarity (Figure 2A and Figure S1 (Supplementary Materials)), accordingly similar synthesis and ligation strategies can be utilized. One notable difference is that LC3A contains one cysteine residue (Cys17), which however is located in the N-terminal region of the protein and as such this residue is ruled out as potential ligation site. The introduction of an alanine to cysteine mutation situated in the middle region of both target proteins would allow an optimal NCL strategy as both the N-terminal and C-terminal peptide would have a similar length and molecular weight (Figure 2A). Typically, such alanine to cysteine mutation sites can be converted back to the native alanine post NCL using reductive desulfurization conditions. The presence of a propargyl moiety at the C-terminus of the protein, however, prevents the use of desulfurization chemistry due to side reactions that compromise the integrity of the propargyl, previously observed by Witting et al. [13]. The alanine to cysteine mutation introduced in the proteins will therefore be permanent, however as reported for Ufm1 [13] and ISG15 [23] is not expected to influence the protein structure or recognition by interacting proteins. In addition, previous work on LC3 that makes use of single point mutants leaving the non-naturally occurring cysteine residue in the sequence did not preclude recognition and proteolysis by the tested proteases ATG4B and RavZ [18,19,20]. A potential junction suitable for ligations was identified at Ala78 and Gln77 for both LC3A and LC3B (Figure 2A), as Ala78 could be replaced by a cysteine to facilitate NCL. This requires a Gln77 thioester N-terminal peptide and a Ala78Cys mutant C-terminal peptide to be prepared. For the N-terminal thioester fragment, the chosen disconnection at glutamine is known to be a challenging amino acid to form a thioester on due to the possibility of self-cyclization after C-terminal activation. To minimize the cyclization of glutamine upon activation, we decided to use the 3,4-diaminobenzoic acid (Dbz) linker in SPPS [24], used successfully by Premdjee et al. on a glutamine residue [25]. As reported, the second amine of the Dbz linker is susceptible to acylation and leads to the accumulation of branched and acetylated peptide products. To prevent this, an orthogonal protecting group, allyloxycarbonyl (alloc), was introduced for SPPS and removed prior to TFA cleavage [26]. We synthesized the N-terminal peptide (peptide 1) uneventfully on Chemmatrix resin loaded with the alloc-protected Dbz linker (Figure 2B and Table S1). Two 2, 4-dimethoxybenzyl (DMB)-dipeptides were incorporated based on an earlier optimization study [21]. As final step in the SPPS, the alloc was removed using Pd chemistry [27]. Next, peptide 2 was cleaved from the resin and treated with NaNO_2_ at pH 3 to convert the Dbz to the corresponding acyl benzotriazole (3) which could be intercepted by MESNa to form the stable LC3B-MESNa thioester (4) (Figure S3) [24]. The N-terminus of the LC3A-thioester (8) was synthesized using a similar protocol (Figure S4).

The synthesis of the C-terminal fragment on 2-chloro trityl chloride (CTC) resin turned out to be challenging and little to no product could be observed using conventional SPPS. Hence, we set out to investigate the cause of the failed synthesis of this peptide with automated fast-flow peptide synthesis (AFPS), capable of monitoring the swelling of the resin during peptide-chain growth. A decrease in resin swelling correlates to aggregation of the growing peptide chain, therefore the problematic regions within the peptide sequence can be observed [28]. A significant decrease in resin swelling was observed after Tyr110, Met111 and Val98 followed by a relative slow increase in swelling for the rest of the synthesis (Figure S2). Based on these results and our earlier reported optimization studies [21], pseudoproline dipeptides were incorporated on position 90 and 95 prior to the challenging regions to prevent the aggregation from occurring (Figure 2 and Table S1). LC3B-peptide 9 was hence prepared on CTC resin and released from the resin by mild acidic cleavage to liberate the C-terminal carboxylic acid while leaving all side chain protecting groups in place. Next, the propargyl moiety was coupled to the C-terminus followed by acid-promoted cleavage of all the protecting groups. Crude peptide 9, however, turned out to be poorly soluble in many solvents including DMSO and denaturing buffers, such as 6 M Gdn. Due to its hydrophobicity, the peptide does not elute from the reverse phase high-performance liquid chromatography (RP-HPLC) column, hence excluding purification by standard RP-HPLC. Investigation of alternative purification methods led to the Belyntic catch-and-release purification method, based on a traceless cleavable linker attached to the N-terminus of the full length peptide [22]. The use of capping during SPPS is essential when using this method to prevent the coupling of the linker to truncated peptides. Release from the resin results in a crude mixture of which only the full-length peptide equipped with the catch-and-release linker can be recovered via an immobilization step on a secondary resin. This protocol was followed step-by-step resulting in peptide 9 in decent purity (Figure S5A–C). Similar solubility difficulties due to the high sequence overlap were observed for the C-terminus of LC3A (peptide 10), which could be solved using the same protocol (Figure S5D–F).

**Figure 3:**
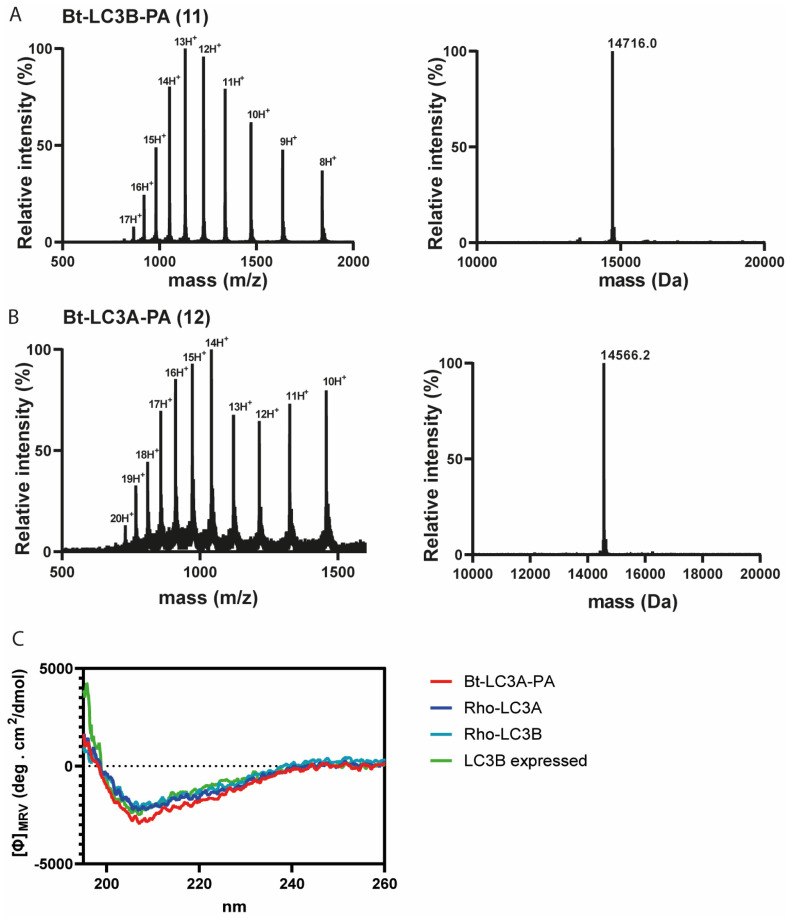
(**A**) Electro-spray ionization (ESI) spectrum of purified Bt-LC3B-propargylamide 11 (left panel) and deconvoluted mass (right panel). Calculated: 14,715.1 Da, observed: 14,716.0 Da, (**B**) ESI spectrum of purified Bt-LC3A-propargylamide 12 (left panel) and deconvoluted mass (right panel). Calculated: 14,565.6 Da, observed: 14,566.2 Da. (**C**) Circular dichroism spectra showing molar ellipticity Φ for recombinant LC3B, synthetic Rho-LC3A and Rho-LC3B and Bt-LC3A- propargylamide probe 11. Dashed line indicates no ellipticity. MRV: mean residue molar ellipticity value; deg: degrees.

Having successfully synthesized the two C-terminal and two N-terminal fragments, attention turned to the assembly of the full LC3A and LC3B proteins using NCL (Figure 2C). To ensure favorable ligation kinetics, typically a high reaction concentration is preferred, however, due to the poor solubility of the C-terminal fragment, high concentrations were not reachable. NCL of fragment 4 with 9 (LC3B) and 8 with 10 (LC3A) were performed at a concentration of 0.5 mM in 6 M Gdn.HCl containing 25 mM of tris(2-carboxyethyl)phosphine and 100 mM MPAA at pH 7 (Figure S6). RP-HPLC purification resulted in both full length Bt-LC3A-propargylamide and Bt-LC3B-propargylamide (Figure 3). Although hydrolysis of the N-terminal thioester fragments was significant, presumably due to the unfavorable dilute NCL conditions, an overall amount of 2.5 and 3.8 mg of purified product could be obtained for Bt-LC3B-propargylamide and Bt-LC3A-propargylamide, respectively. The final challenge was folding and characterization of the synthetic proteins using stepwise dialysis from 6 M Gdn buffer to phosphate buffered saline (PBS). Next, the synthetic proteins LC3B-propargylamide (11) and LC3A-propargylamide (12) were characterized by LC-MS (Figure 3A), circular dichroism (CD) measurements (Figure 3B) and SDS-PAGE analysis (Figure S8). As expected, based on the literature precedents of correctly folded Ub-like proteins containing non-naturally occurring cysteine residues, proper folding was confirmed using CD analysis that showed similar results for expressed LC3B, synthetic Rhod-LC3A, Rhod-LC3B [21] containing the native peptide sequence and synthetic Bt-LC3A-propargylamide containing the alanine to cysteine mutation (Figure 3C), indicating that neither the cysteine to alanine mutant, the N-terminal propargylamide modification nor C-terminal biotin modification affected the overall LC3 fold.

**Figure 4:**
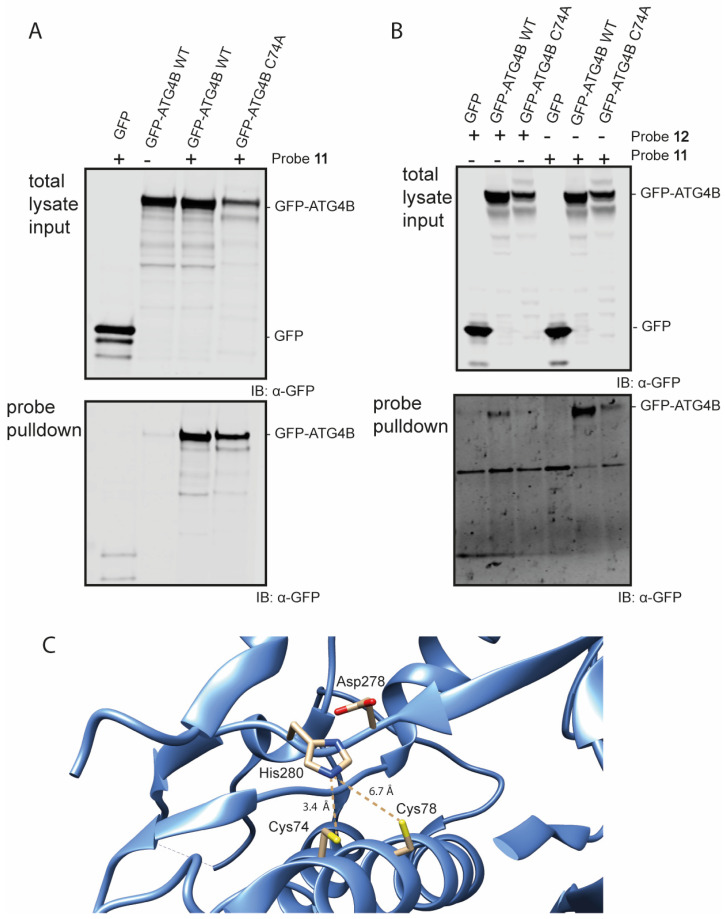
Western blot analysis of the pull-down of GFP-Atg4B and GFP-Atg4B C74A from cell lysate (**A**) using probe 11 and mild washing conditions. (**B**) using probes 11 and 12 using harsh washing conditions. (**C**) Crystal structure of the active site of Atg4B (PDB: 2CY7). Cys74 has a distance of 3.4 Å to the His280 residue and Cys78 has a distance of 6.7 Å to the His280 residue.

With these ABPs in hand, their reactivity towards the Atg4B cysteine protease was tested in an in vitro assay. Firstly, we assessed Bt-LC3B-propargylamide (11) in lysate of HEK293T cells transiently overexpressing GFP-tagged Atg4B or a catalytic inactive mutant GFP-tagged Atg4B C74A. The cells were lysed and incubated with probe 11 at 37 °C, followed by a pull-down on the biotin attached to the LC3B-probe using streptavidin beads (Figure 4 and Figure S9). Visualization by Western blotting using anti-GFP antibody revealed that Atg4B WT is efficiently pulled-down by probe 11 (Figure 4A bottom panel lane 3), indicating a strong interaction between LC3B and ATG4B. An interesting finding is that the probe is also capable of pulling down the catalytic inactive mutant (Figure 4A, bottom panel lane 4). This indicates that either the protein–protein interaction between Atg4B and LC3B (including the catalytic inactive mutant) is very strong or the propargyl probe is able to form a covalent bond with the active site cysteine in the ATG4B wild type and potentially another residue (probably cysteine) in the Atg4B Cys74Ala protein.

The latter seems unlikely, since propargyl probes are considered to be inert and do not react with an excess of thiol or non-active site cysteine residues [14,29]. To investigate this further, a second pull-down experiment followed by harsh washing conditions (buffer containing 2% SDS) of the beads was performed that should wash out all non-covalent interactions. Even after stringent washing, Atg4B WT pull-down was still observed with either Bt-LC3A-propargylamide or Bt-LC3B-propargylamide, indicating formation of a covalent complex between probe and protease (Figure 4B, bottom panel lanes 2 and 5). The catalytic inactive mutant was also still pulled-down, however to a lesser extent than observed previously using the less stringent washing steps (Figure 4B, bottom panel lanes 3 and 6). Inspection of the amino acid sequence and crystal structure of Atg4B revealed an additional cysteine (Cys78) residue in close proximity to the active site cysteine (Cys74) (Figure 4C) [30]. Although the alignment and distance of Cys78 in the catalytic triad of the protease is less favorable then Cys74, we hypothesize that Cys78 is able to complement the incompetent active site in the ATG4B C74A mutant rendering it partially reactive towards the Bt-LC3B-propargylamide and Bt-LC3A-propargylamide probes.

## 4. Discussion

Activity-based protein profiling has proven to be a powerful strategy for monitoring both the enzyme activities involved in ubiquitin-like (Ub(l)) conjugation and deconjugation cascades and a viable approach to discover novel Ub(l)-processing enzymes and study their modes of action [14,31,32,33]. We envision that the LC3-propargylamide probes presented here can be applied similarly in the study of other enzymatic activities in the LC3 cascade. In addition to expression-based semi-syntheses and linear SPPS routes, we here introduce an effective NCL approach to LC3A and LC3B proteins allowing the simultaneous introduction of N-terminal and C-terminal modifications such as fluorophores, affinity tags or warheads. The described methodology opens the way for the synthesis of additional LC3 probes and reagents, in analogy to the toolbox present for Ub and other Ub-like proteins. Our observations that both LC3A-propargylamide and LC3B-propargylamide bind to the catalytic active and inactive mutant of Atg4B warrants further research. Although the non-active site mediated binding (C74A mutant) decreases when harsh washing conditions are applied, still significant residual enrichment with the LC3B-propargylamide can be observed. We speculate that Cys78 is able to partially restore the incomplete active site in the Cys-to-Ala mutant and rescue the activity towards the LC3-probes. In light of this hypothesis, it is important to consider the placing of the warhead in our probes as ATG4B cleaves the amide bond between Gly120 and PE. In our probes the propargyl moiety is coupled to the C-terminus of Gly120. Other Ub(l)- propargylamide probes typically have the propargyl warhead substituting the C-terminal glycine and hence are ΔG-propargylamide probes. The effectiveness of our probes and potential side reactivity with Cys78 might be influenced by this additional length and potential partial misalignment in the active site of the protease. Furthermore, one could reason that the propargyl moiety itself serves as a mimic for the conjugated PE and hence is a suitable substrate for Atg4B. The active site of ATG4B might be more flexible then for DUBs and hence accommodate the LC3-propargylamide probes, which is also demonstrated by the efficient pull-down by both probes. Future variations on placing the warhead in different positions and modifying the nature of the warhead part including PE variants might give further insights into the protease’s functioning.

## 5. Conclusions

In conclusion, we present an NCL approach for the chemical synthesis of LC3 activity-based covalent probes, that allows for modification of both N- and C-termini. The addition of this methodology to previously reported routes and avenues towards small molecule modulators of the autophagy pathway [34] further opens the way to the expansion of the LC3 toolbox. A combination of multiple approaches thus can be valuable to further interrogate the LC3 conjugating and deconjugating enzymes, that might prove useful in obtaining new insights in the molecular details governing autophagy.

## Data Availability

Data is available in the accompanying Supplementary Materials.

## Supplementary Materials

The following supporting information can be downloaded at: https://www.mdpi.com/article/10.3390/biomedicines11030884/s1, Supporting Information describing details on synthesis of peptides and cell culture.
