# Supplementary material for: Total Chemical Synthesis of LC3A and LC3B Activity-Based Probes
Source: Biomedicines. 2023 Mar 13;11(3):884. doi: 10.3390/biomedicines11030884 (PMC10045837; doi:10.3390/biomedicines11030884)
Supplement: Supplementary file 1 [file biomedicines-11-00884-s001.zip › biomedicines-2218800-supplementary.pdf]

Total Chemical Synthesis of LC3A and LC3B activity-based probes

Yara Huppelschoten[1,2], Jens Buchardt [2,3], Thomas E. Nielsen[2], Aysegul Sapmaz[1] and Gerbrand J. van der Heden van Noort[1*]

[1] Dept. Cell and Chemical Biology, Leiden University Medical Centre, Leiden, The Netherlands

[2] Global Research Technologies, Novo Nordisk A/S, Novo Nordisk Park, DK-2760 Måløv, Denmark

[3] current address: CMC API Development, Novo Nordisk A/S, DK-2880 Bagsværd, Denmark

e-mail: gvanderheden@lumc.nl

Peptide sequences

```
                 ....:....| ....:....| ....:....| ....:....| ....:....| ....:....|
LC3B             ------MPSE KTFKQRRTFE QRVEDVRLIR EQHPTKIPVI IERYKGEKQL PVLDKTKFLV  54
LC3A             ------MPSD RPFKQRRSFA DRCKEVQQIR DQHPSKIPVI IERYKGEKQL PVLDKTKFLV  54
LC3C             MPPPQKIPSV RPFKQRKSLA IRQEEVAGIR AKFPNKIPVV VERYPRETFL PPLDKTKFLV  60
GABARAP_1        --------MK FQYKEDHPFE YRKKEGEKIR KKYPDRVPVI VEKAPKAR-V PDLDKRKYLV  51
GABARAP_         --------MK FVYKEEHPFE KRRSEGEKIR KKYPDRVPVI VEKAPKAR-I GDLDKKKYLV  51
GABARAP_2        --------MK WMFKEDHSLE HRCVESAKIR AKYPDRVPVI VEKVSGSQ-I VDIDKRKYLV  51
                              :*: :.:   *  :   **  :.* ::**: :*:      :   :** *:**

LC3B             PDHVNMSELI KIIRRRLQLN ANQAFFLLVN GHSMVSVSTP ISEVYESEKD EDGFLYMVYA  114
LC3A             PDHVNMSELV KIIRRRLQLN PTQAFFLLVN QHSMVSVSTP IADIYEQEKD EDGFLYMVYA  114
LC3C             PQELTMTQFL SIIRSRMVLR ATEAFYLLVN NKSLVSMSAT MAEIYRDYKD EDGFVYMTYA  120
GABARAP_1        PSDLTVGQFY FLIRKRIHLR PEDALFFFVN N-TIPPTSAT MGQLYEDNHE EDYFLYVAYS  110
GABARAP          PSDLTVGQFY FLIRKRIHLR AEDALFFFVN N-VIPPTSAT MGQLYQEHHE EDFFLYIAYS  110
GABARAP_2        PSDITVAQFM WIIRKRIQLP SEKAIFLFVD K-TVPQSSLT MGQLYEKEKD EDGFLYVAYS  110
                 *..:.: ::   :** *: *  . .*::::*:    :   * . :.::*.. :: ** *:*:.*:

LC3B             SQETFG 120
LC3A             SQETFG 120
LC3C             SQETFG 126
GABARAP_1        DESVYG 116
GABARAP          DESVYG 116
GABARAP_2        GENTFG 116
                 .:..:*
```

Figure S1. Alignment of human LC3A, LC3B, LC3B, GABARAP, GABARAP1 and GABARAP2. * indicates amino acid positions that have a fully conserved residue, : indicates conservation of amino acids with strongly similar properties, . indicates conservation of amino acids with weakly similar properties.

Table S1. Underlined dipeptide sequences were coupled as the respective pseudoproline dipeptides (in red) and 2, 4-dimethoxybenzyl (DMB)-dipeptides (in blue).

Segment ID	SPPS sequence
LC3B NTerm (peptide 4)	MPSEKTFKQRRTFEQRVEDVRLIREQHPTKIPVIIERYKGEKQLPVLDKTKFLVPDHVNMSELIKIIRRRLQLNANQ-Dbz-G
LC3A NTerm (peptide 8)	MPSDRPFKQRRSFADRCKEVQQIRDQHPSKIPVIIERYKGEKQLPVLDKTKFLVPDHVNMSELVKIIRRRLQLNPTQ-Dbz-G
LC3B CTerm (peptide 9)	CFFLLVNGHSMVSVSTPISEVYESEKDEDGFLYMVYASQETFG
LC3A CTerm (peptide 10)	CFFLLVNQHSMVSVSTPIADIYEQEKDEDGFLYMVYASQETFG

Aggregation scan of peptide 9 using automated fast flow synthesis

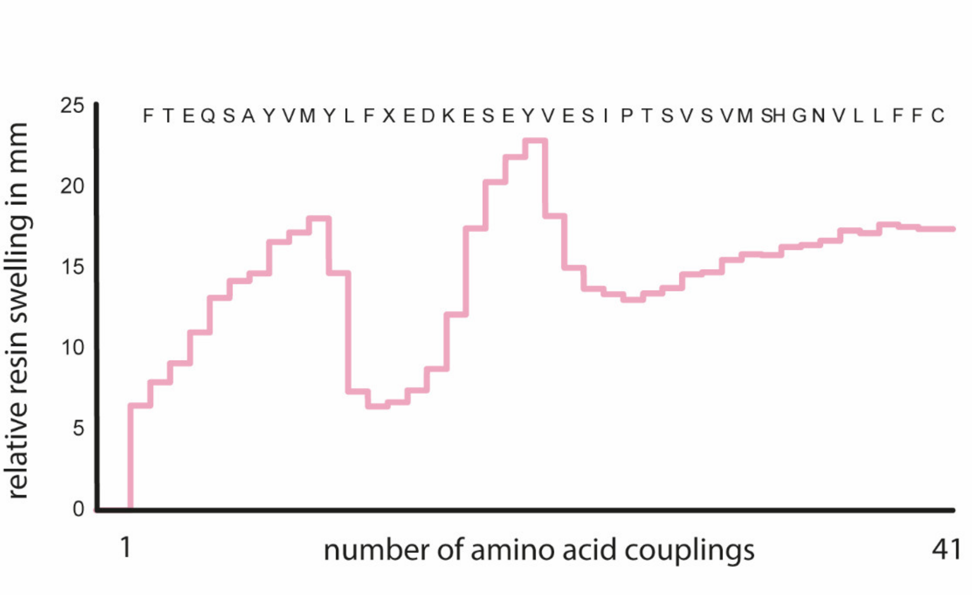

Figure S2. Aggregation scan of the peptide 9. Y-axes displays resin swelling in mm relative to initial volume of the resin, x-axes displays the number of amino acid couplings that were performed. The sequence of peptide 9 is shown above the graph.

General procedures

Materials and solvents

Reagents were obtained from Sigma-Aldrich of the highest available grade and used without further purification. Standard Fmoc-protected amino acid derivatives were used and purchased from Gyros Protein Technologies (Gyros Protein Technologies, Uppsala, Sweden) unless mentioned otherwise. Fmoc-Cys(acetamidomethyl (Acm))-OH and resins for SPPS were obtained from Novabiochem (Merck Millipore, Darmstadt, Germany), Apigenex S.r.o (Cesko, Czechia) and PCAS Biomatrix (Saint-Jean-sur-Richelieu, Canada). Pseudoproline dipeptides were obtained from Corden Pharma GmbH (Plankstadt, Germany) or Bachem (Bubendorf, Switserland). Iso-acyl dipeptides were obtained from AAPPTec (Louisville, Kentucky, USA). Solvents for SPPS were obtained from Biosolve (Valkenswaard, The Netherlands). VA-044 was procured from Wako Pure Chemical Corporation (Neuss, Germany). Oxyma Pure® was purchased from Gyros Protein Technologies. HPLC grade acetonitrile was obtained from Merck (Darmstadt, Germany).

Analytical methods

LC-MS conditions

LC-MS measurements were performed on a Waters Acquity UPLC H Class system, Waters Xevo G2-XS QTof (Waters Corp., Milford, MA, USA) with a Waters Acquity BEH 300 Å, C4, 1.7 µm, 2.1 mm x 50 mm (0.4 mL/min). Samples were run at 60 °C using 3 mobile phases: A = 0.1 % formic acid in deionized water, B = 0.1 % formic acid in acetonitrile and C = 0.01 % TFA in dionized water with a gradient of 5 to 25% B over 1 min, 25 to 65 % B over 6 min followed by 65 to 95 % B over 0.5 min maintaining a composition of 5% C throughout. Data processing was performed using Waters MassLynx Mass Spectrometry Software V4.2 (deconvolution with MaxEnt I function).

Solid Phase Peptide Synthesis (SPPS)

Loading Dawson linker

Chemmatrix rink amide resin (0.69 mmol/g) (Novabiochem, Merck Millipore, Darmstadt, Germany) was loaded with Fmoc-Gly-OH (0.3 equivalent) as described in general methods, followed by capping and Fmoc deprotection. The resin was washed with DMF (3 x 20 mL) and Fmoc-Dbz-OH was coupled as described in the general procedures and reacted for 16 hr. The reaction mixture was drained and the resin washed with DMF (6 x 20 mL) and DCM (3 x 20 mL). Next, allyl chloroformate (350 mM) and DIPEA (1 equivalent to resin loading) in anhydrous DCM were added and reacted for 16 hr. Followed by washing the resin with DMF (6 x 20 mL) and DCM (3 x 20 mL), the resin was dried in vacuo overnight before use.

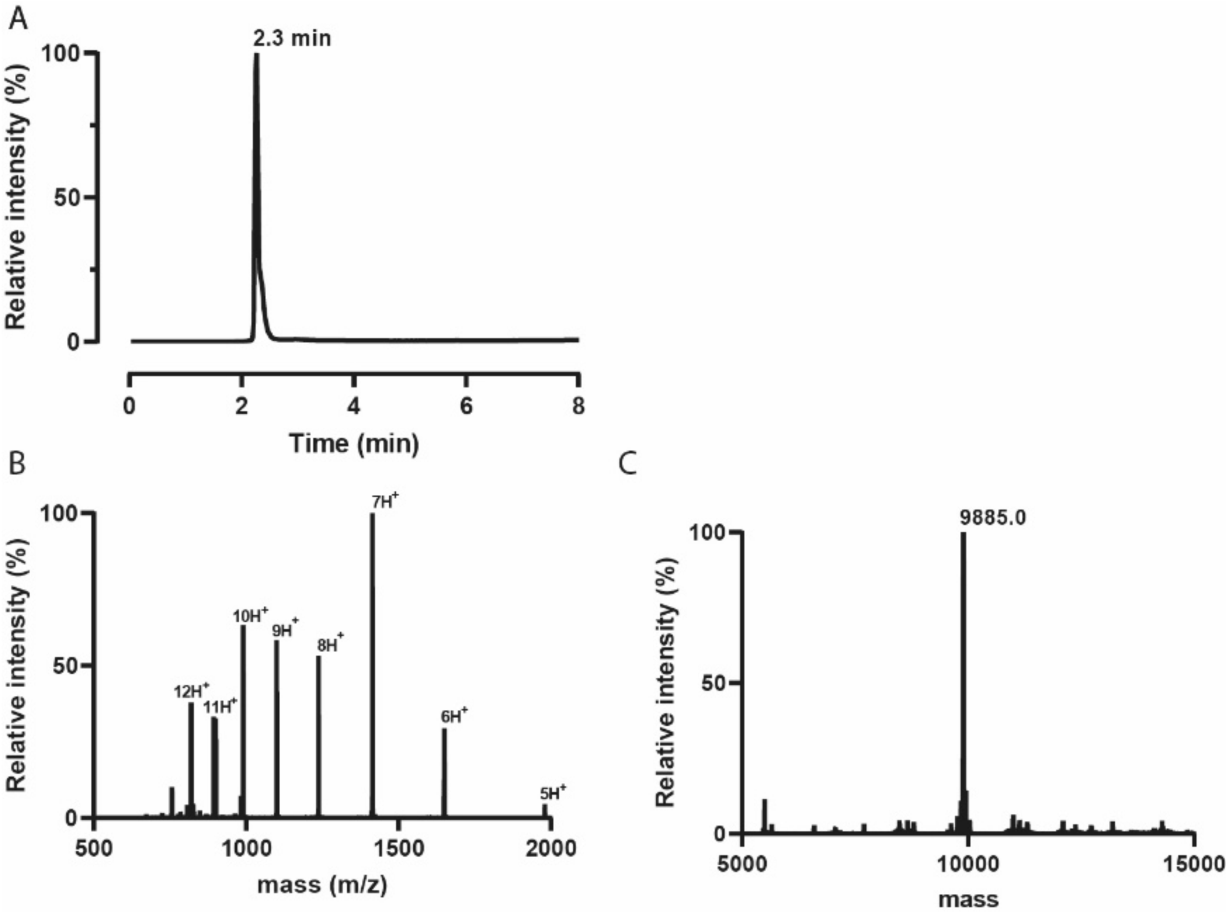

Figure S3. Analysis of peptide 4. **A.** Total ion spectrum (LC-MS method C4) of 4, Rt: 2.3 min, **B.** ESI spectrum of purified peptide 4, **C.** Deconvoluted mass of purified peptide 4, mass calculated: 9884.8 Da, observed: 9885.0 Da.

Synthesis of peptide 8

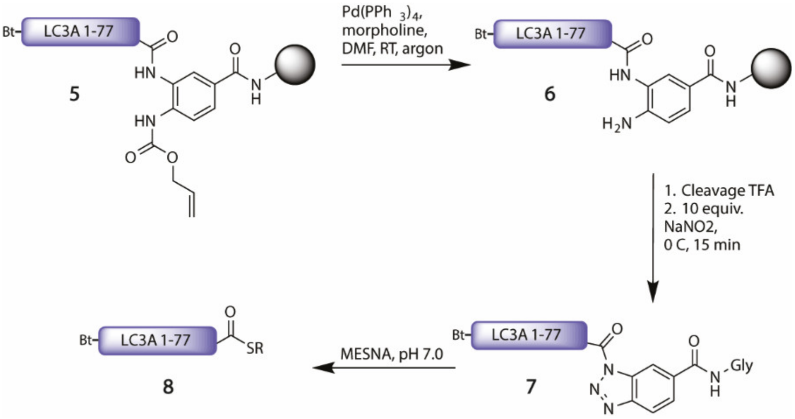

The synthesis was performed following the procedure described for automated SPPS using Chemmatrix resin preloaded with Dbz(alloc) (0.44 g, 0.23 mmol/g). Alloc deprotection was achieved by swelling the resin in dry DCM and adding $Pd(PPh_3)_4$ (43 mg, 0.03 mmol, 0.35 equivalent) and morpholine (176 µL, 2.1 mmol, 20 equivalents). The reaction proceeded for 4 hr and afterwards the resin was washed with DMF (3x) and DCM (3x). The peptide was cleaved from the resin according to the general procedures. Peptide 6 was dissolved in 6 M Gdn.HCl pH 3.0 (1 mM final concentration) and 1 M $NaNO_2$ in deionized water (1 mL, 1 mmol, 10 equivalents) was added and stirred for 5 min at 0 °C. The reaction was warmed to room temperature and MESNa (1.34 g, 8 mmol, 80 equivalents) in 6 M Gdn.HCl, 0.2 M phosphate pH 7.0 was added. The pH was adjusted to pH 7.0 and the solution was stirred for 20 min before purification by preparative HPLC using a Gemini® ® (Phenomenex Inc., Torrance, CA, USA) 110 Å, C18, 5 µm, 30 mm x 250 mm column (25 to 35% B over 20 min, 30 mL/min). Lyophilization afforded peptide 8 as a white solid (33.12 mg, 3.3 % yield). Calculated mass: 9717.3 Da, observed: 9716.8 Da.

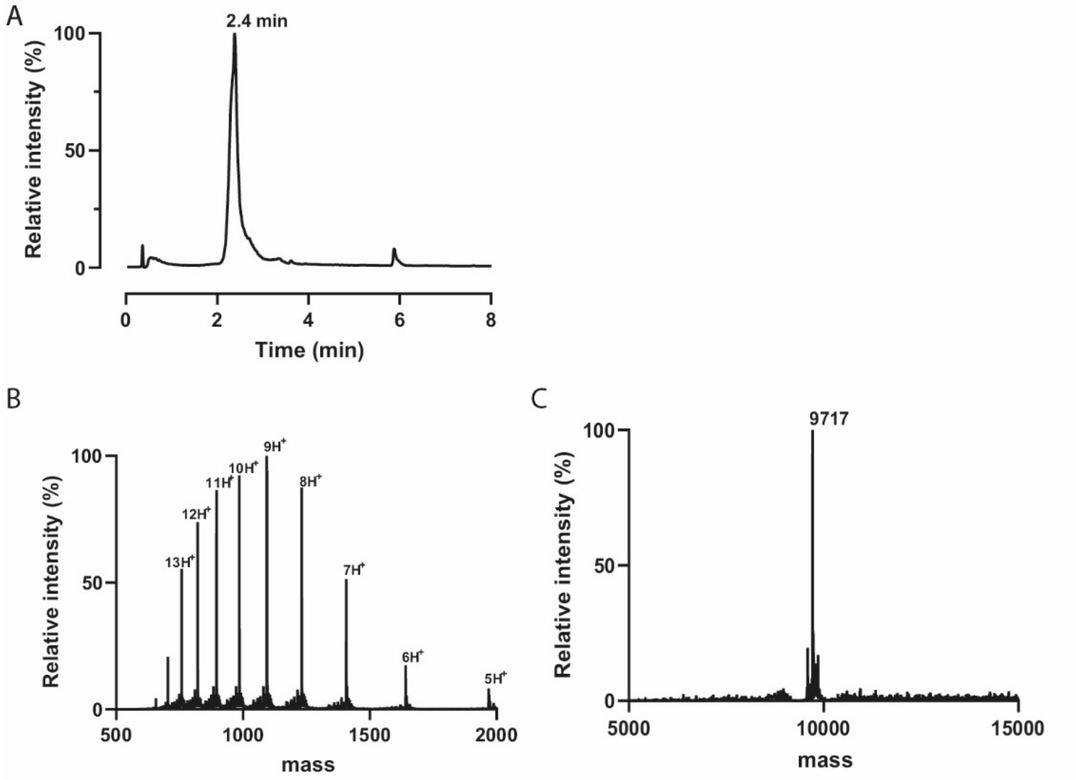

Figure S4. Analysis of peptide 8 **A.** Total ion spectrum (LC-MS method C4) of 8, Rt: 2.4 min, **B.** ESI spectrum of purified 8, **C.** Deconvoluted mass of purified 8, mass calculated: 9717.3 Da, observed: 9717.0 Da.

Synthesis of peptide 10

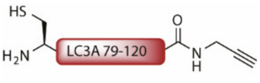

10

The synthesis was performed following the procedure described for automated SPPS using 2-chlorotrityl resin preloaded with Fmoc-Glycine (0.37 g, 0.42 mmol/g). As final step the Belyntic Peptide-Easy-Clean (PEC) linker was coupled according to manufacturer's procedure.[1] The protected polypeptide is detached from the resin by treatment with HFIP/DCM (1:3), 3x for 15 min. All filtrates were combined and concentrated under reduced pressure. Followed by co-evaporation of the protected protein by DCE. Subsequently, the protected protein was dissolved in DCM and propargyl amine (4 equivalents, 39 µL, 0.62 mmol) and DIPEA (2 equivalents, 54 µL, 0.31 mmol) were added and reacted for 16 hr. The solvents were removed *in vacuo* and the protecting groups were cleaved according to the general procedures. The crude peptide was purified by following the procedure from Belyntic[1] followed by lyophilization to afford peptide 10 as a white solid (8.24 mg, 1.1 % yield).

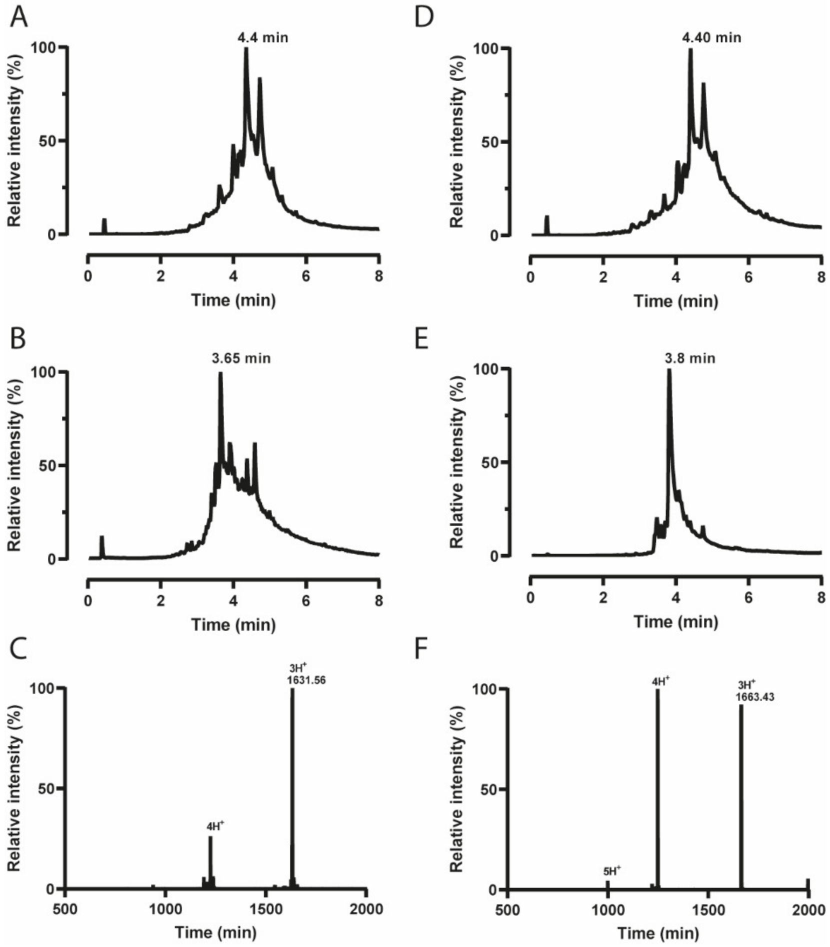

Figure S5. Analyses of peptide 9 and 10. **A.** Total ion spectrum (LC-MS method C4) of crude 9, Rt 4.4 min, **B.** Total ion spectrum (LC-MS method C4) of purified 9, Rt 3.6 min, **C.** Calculated Mass (average isotope composition) of purified 9: 4891.23 Da; $[M + 3H]^{3+}$: 1631.41. Observed: 4891.68 Da; $[M + 3H]^{3+}$: 1631.56, **D.** Total ion chromatog (LC-MS method C4) of crude 10, Rt 4.4 min, **E.** Total ion spectrum (LC-MS method C4) of purified

10, Rt 3.8 min, **F.** Calculated Mass (average isotope composition) of purified 10: 4987.31 Da; $[M + 3H]^{3+}$: 1663.44, $[M + 4H]^{4+}$: 1247.83. Observed: 4987.29 Da; $[M + 3H]^{3+}$: 1663.43, $[M + 4H]^{4+}$: 1247.83.

Assembly of LC3B and LC3A

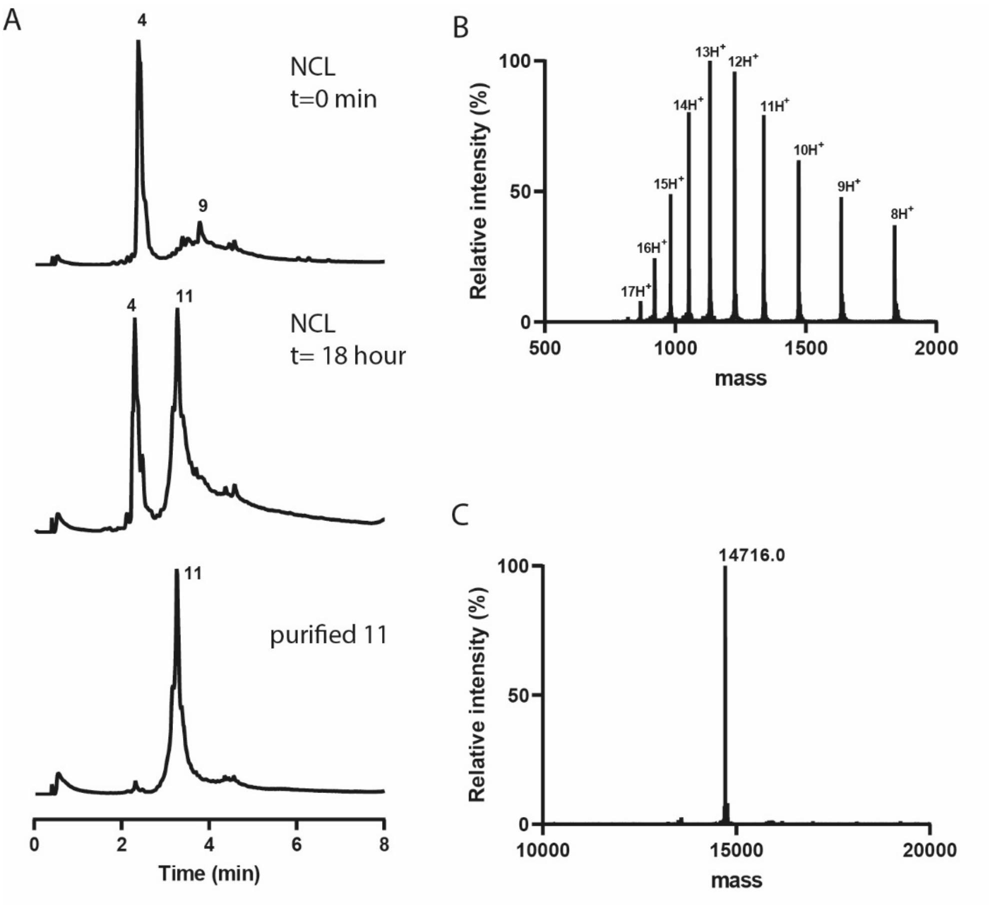

Figure S6. Analysis of Biotin-LC3B-PA 11. **A.** Total ion spectrum (LC-MS method C4) of native chemical ligation reaction between peptide 4 and peptide 9 towards 11 at 0 min, 18 hr and after purification, **B.** ESI spectrum of purified peptide 11, **C.** Deconvoluted mass of purified peptide 11, mass calculated: 14715.1 Da, observed: 14716.0 Da.

Biotin-LC3A-PA (12)

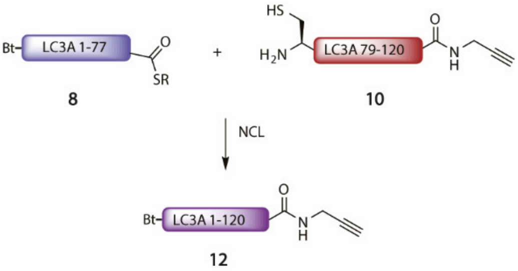

Peptide 8 (11.74 mg, 0.012 mmol) and peptide 10 (10.43 mg, 0.002 mmol) were dissolved in 6 M Gdn.HCl/0.2 M NaH_2PO_4, pH 7.2 at a final concentration of 1 mM. MPAA and TCEP were added from a 1 M stock in deionized water to a final concentration of 100 and 25 mM. Then pH was adjusted to 7.0 and the reaction was shaken for 16 hr at 37 °C upon which LC-MS analysis showed that the reaction was complete. Followed by purification by preparative HPLC using a Gemini® 110 Å, C4, 5 µm, 10 mm x 250 mm column (25 to 45% B over 20 min, 5 mL/min). Lyophilization afforded peptide 12 as a white solid (3.8 mg, 2.2 % yield).

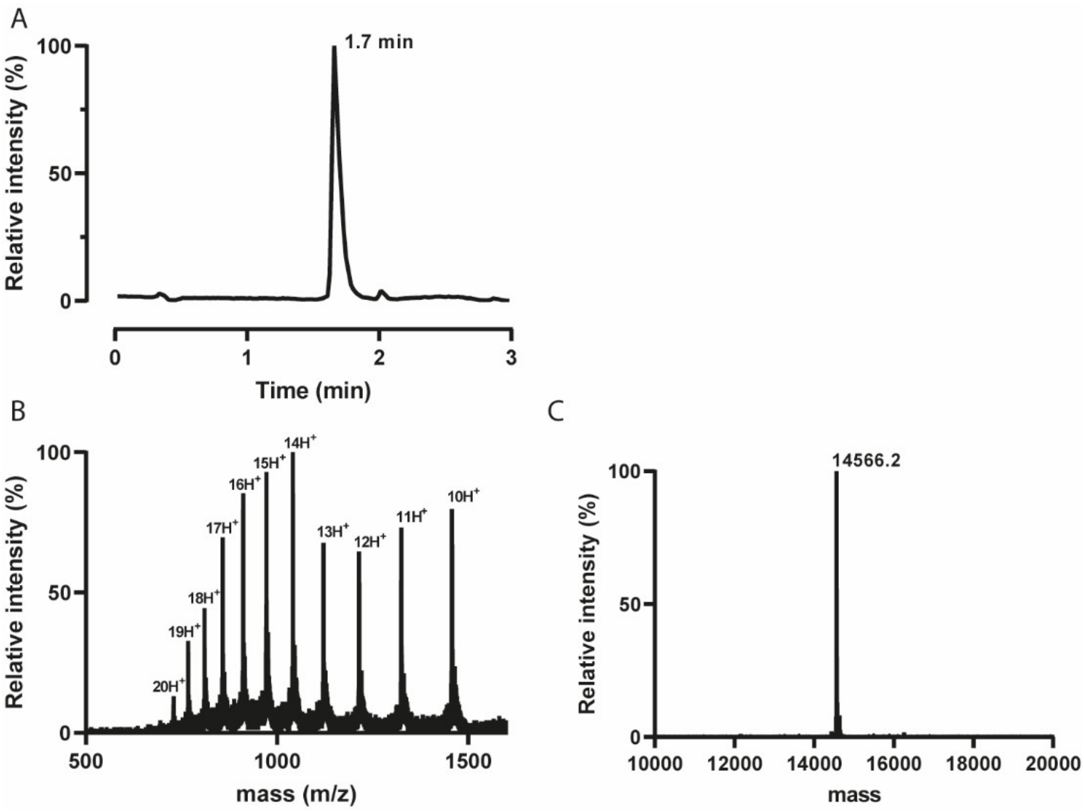

Figure S7. Analysis of Biotin-LC3A-PA 12 **A.** Total ion spectrum of peptide 12, Rt: 1.7 min, **B.** ESI spectrum of purified peptide 12, **C.** Deconvoluted mass of purified peptide12, mass calculated: 14565.6 Da, observed: 14566.2 Da.

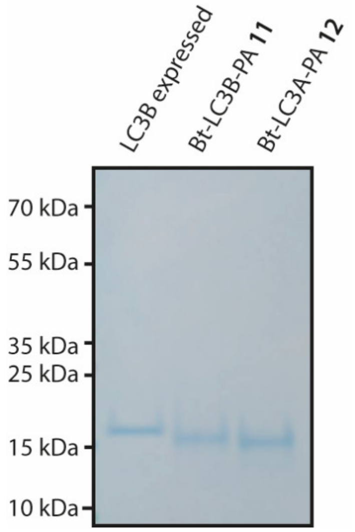

Figure S8. SDS-PAGE analysis of Bt-LC3B-propargylamide 11 and Bt-LC3A-propargylamide 12 in comparison to expressed wild type LC3B. Coomassie stain.

Circular dichroism

CD measurements were performed using a Jasco (Jacso Inc. Easton, MD, USA) 1500 spectropolarimeter at concentrations of 0.1 mg/mL in PBS, pH 7.4, concentrations were measured using a NanoDrop spectrophotometer at A280 (calculated extinction coefficient of 5960 $cm^{-1}M^{-1}$). Reference LC3B was obtained from Abcam (ab103506). Measurements between 250 and 190 nm were taken using a quartz cuvette with a path length of 0.02 cm. In total, 8 cumulative measurements were made and the average was calculated and plotted using GraphPad PRISM.

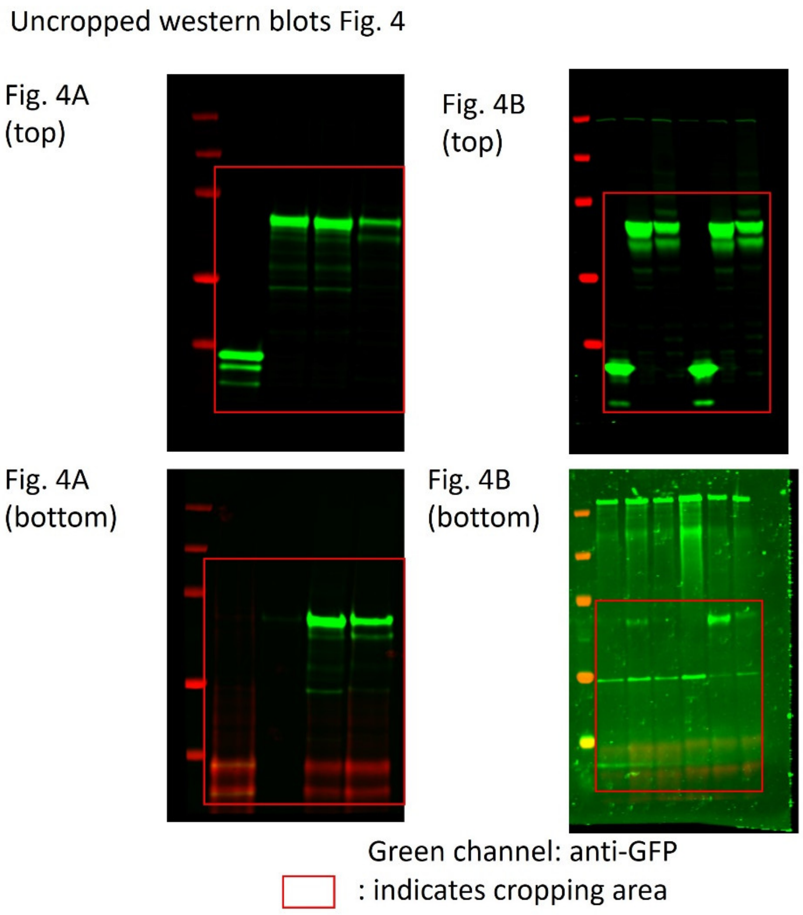

Figure S9. Uncropped Western blots indicating the cropping areas used to prepare Figure 4.

References

[1] Manufacturers protocol can be found on: https://www.gyrosproteintechnologies.com/peptides/products/purepep-easyclean-starter-kit
